# Comments: The need to standardize use of the newly deceased in medical trainings

**DOI:** 10.6061/clinics/2021/e2991

**Published:** 2021-06-07

**Authors:** Seyed Ali Enjoo

**Affiliations:** Medical Ethics Department, School of Traditional Medicine, Shahid Beheshti University of Medical Sciences, Tehran, Iran

Dear Editor,

I have read the article titled “The need to standardize use of the newly deceased in medical trainings” published in Clinics by Cabar FR et al. (1) with avid interest. The article provides information that would be useful for activists and researchers working in the fields of medical ethics and medical education. The implications of the brilliant issues highlighted by Dr. Cabar could be discussed further. In a similar field study, participants were asked two additional questions to evaluate their moral distress and feelings as well as the usefulness of educational training based on the use of the bodies of newly deceased individuals. Further, I would suggest that a more comprehensive evaluation should be performed by asking the participants about their willingness to repeat the training involving the use of the bodies of recently deceased individuals (2). As specified by Moore, despite the trainers’ and trainees’ apprehension or other negative feelings toward educational training programs that employ dead bodies (3), this approach of using dead bodies for training the medical personnel could be perceived as a strategy to save human lives, thereby ensuring that more humans do not become the subjects of medical education upon their death. Moreover, this approach could be considered as “teaching without harm,” as also mentioned by Rajagopal AS and Champney TH (4).

Another aspect worthy of concern is potential cases where consent is lacking- the probability of scandal and even lawsuits associated with the disclosure of the training methods. In the absence of consent from the newly deceased—when he/she was alive and competent or from his/her surrogate decision-makers—some educational procedures could anger the patient’s family, resulting in the filing of lawsuits. Specifically, although some educational procedures such as endotracheal intubation may not easily leave a mark on the bodies, procedures such as tracheostomy or chest tube insertion, which leave pronounced imprints on dead bodies could result in litigation and resentment.

Because of the importance of proper and high quality medical education, efforts to improve the cultural acceptance of this teaching method by highlighting the life-saving aspect of the approach could resolve the aforementioned problems. Mass media could be instrumental in this process. Further, wills or other legal formalities should be considered, such as advanced directives to obtain consent prior to a patient’s death.

I agree with the authors that guidelines and codes of conduct for improving medical education—e.g., the existing guidelines for medical research—should be established at both the national and local levels. I would like to commend the Clinics journal for publishing such an impactful article.
